# Triple-Negative Sjogren’s Syndrome and Recurrent Pneumothorax: An Uncommon Presentation of Autoimmune Disease

**DOI:** 10.7759/cureus.26636

**Published:** 2022-07-07

**Authors:** Ziryab Imad, Yassin A Abdalla, Salih B Hamza, Mohammed E Abubakr, Fathelrahman H Elneel, Fatima M Mustafa, Sami A Abdalgadir, Jimmy William

**Affiliations:** 1 Rheumatology, Haj Elsafi Teaching Hospital, Khartoum, SDN; 2 Internal Medicine, University of Bahri, Khartoum, SDN; 3 Rheumatology, Ziryab Research Group, Khartoum, SDN; 4 Internal Medicine, Omdurman Islamic University, Omdurman, SDN; 5 Thoracic Surgery, Al Shaab Teaching Hospital, Khartoum, SDN; 6 Internal Medicine, Al Shaab Teaching Hospital, Khartoum, SDN; 7 Radiology, Omdurman Military Hospital, Omdurman, SDN; 8 Internal Medicine, Sligo University Hospital, Sligo, IRL

**Keywords:** triple negative ss, recurrent pneumothorax, ss-a & ss-b, extra-articular manifestations, primary sjogren’s syndrome

## Abstract

Sjogren's syndrome (SS) is a chronic exocrinopathy caused by lymphocytic infiltration and is associated with numerous manifestations and morbidities. We discuss a case of a 60-year-old female who presented to the Acute Medical Assessment Unit complaining of progressive shortness of breath for one month, not associated with chest pain or lower limb swelling. She also reported joint pain involving both wrists and proximal interphalangeal (PIP) joints, oral dryness, hair loss, and numerous tongue ulcerations. Blood workup revealed triple-negative SS, negative rheumatoid factor, anti-SSA and anti-SSB, a high erythrocyte sedimentation rate (ESR), and antinuclear antibody (ANA) titer of 640. A diagnosis of SS was made. Nevertheless, her CT chest showed massive left-sided pneumothorax; subsequently, a chest tube was urgently inserted. The chest tube was removed two days later with complete resolution on chest X-ray (CXR). However, one week later, she presented with a recurrent pneumothorax that persisted and required surgical intervention that led to complete recovery afterward. Pneumothorax is an extremely rare but potentially unfavorable complication related to SS, with only two cases reported in the literature so far and usually associated with underlying lung pathology.

## Introduction

Sjogren's syndrome (SS) is a chronic autoimmune exocrinopathy characterized by lymphocytic infiltration of the glandular and extraglandular organs [1]. SS is frequently associated with numerous respiratory complications, including tracheobronchial sicca, small airway disease, various patterns of interstitial lung disease, lymphoproliferative disease, pulmonary hypertension, pleural involvement, and pulmonary amyloidosis [2]. However, the literature has published only two cases of spontaneous pneumothorax associated with SS [3,4]. We report a rare case of massive unilateral pneumothorax associated with SS. Another remarkable aspect of this case is the recurrence of the pneumothorax after the initial complete resolution with no other etiology identified.

## Case presentation

**Figure 1:**
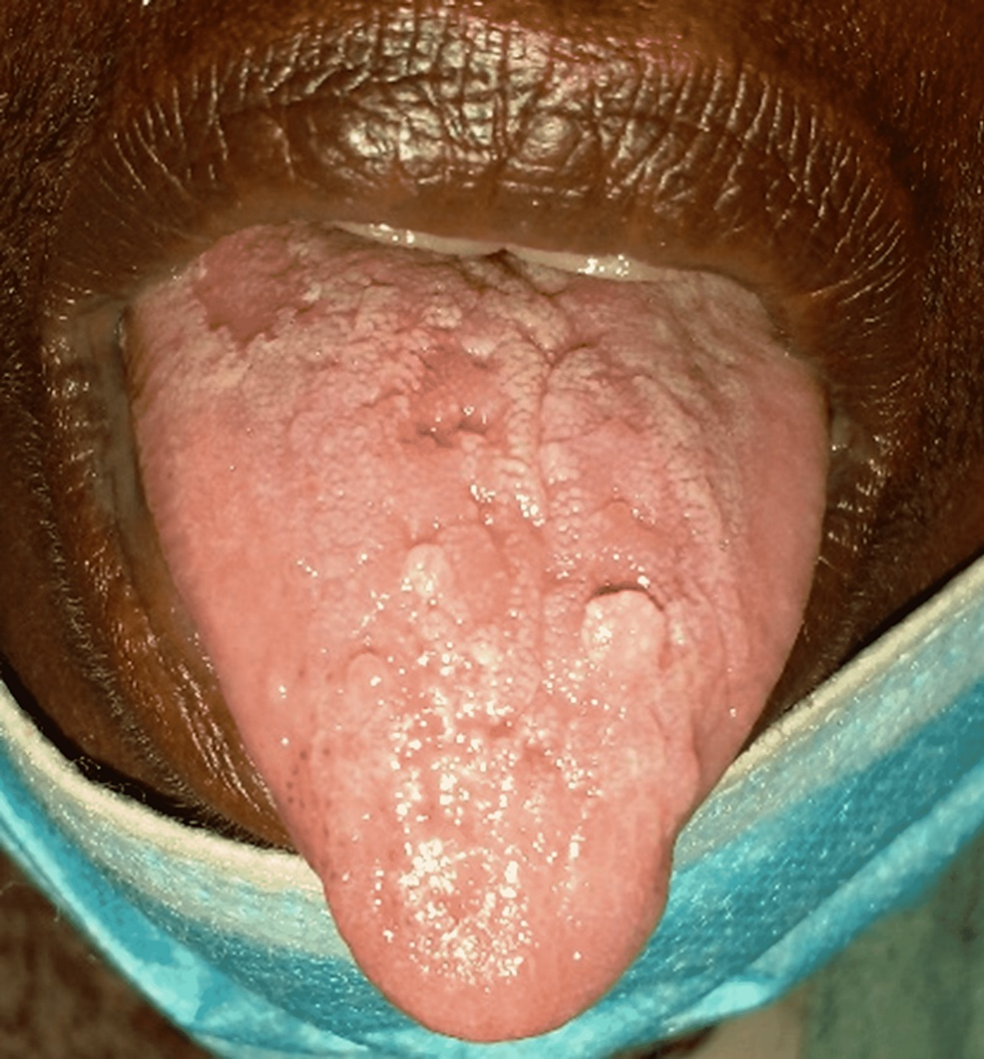
Tongue ulcerations

A 60-year-old Sudanese female, nonsmoker/non-alcoholic, with no significant past medical history other than well-controlled hypertension for eight years, was referred from White Nile state with progressive shortness of breath and palpitation for one month. Her dyspnea had been initially associated with moderate exertion; however, it had progressed to a point where even routine daily activities were affected. It was not associated with orthopnea, paroxysmal nocturnal dyspnea, chest pain, or swelling of the lower limb. She reported a dry cough for the same duration; nevertheless, she had no weight loss, fever, or loss of appetite. The patient also described bilateral joint pain involving the wrist and proximal interphalangeal (PIP) joint, sparing the distal interphalangeal (DIP) joint. There was also oral dryness with tongue ulcers (Figure 1), dryness of the eyes, and hair loss but no skin rash or genital ulcers. Her systematic review was unremarkable apart from chronic constipation. She had been diagnosed with hyperthyroidism 15 years ago but was not on long-term medication apart from amlodipine 5 mg and aspirin 75 mg. Physical examination revealed oral mucosal dryness with tongue ulceration, but no joint deformity, skin tightness, organomegaly, or lymphadenopathy. Blood workup revealed the following findings: unremarkable full blood count (FBC), elevated erythrocyte sedimentation rate (ESR), positive antinuclear antibody (ANA) global with nucleolar/fine cytoplasmic granule pattern with a titer of 1/640 yet negative for all ANA parameters including anti-dsDNA, Scl-70, SS-A, and SS-B. Rheumatoid factor and anti-CCP were also negative. Schirmer's test was positive being 4 bilaterally and ocular staining of 6, and hence a diagnosis of triple-negative primary SS (pSS) was made. Consequently, the patient was commenced on prednisolone for three months with gradual tapering and azathioprine 50 mg once daily.

**Figure 2:**
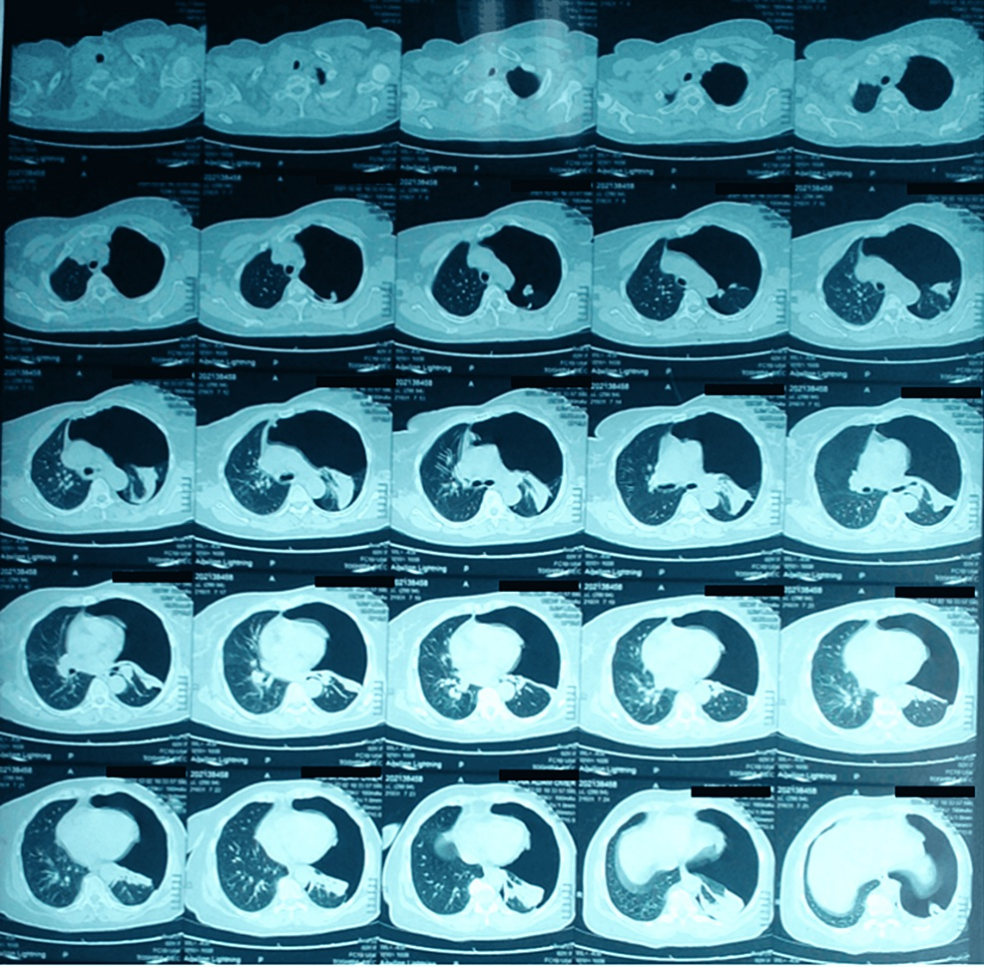
CT chest taken before the first admission showing massive left-sided pneumothorax CT: computed tomography

A CT chest was performed, which revealed a large left pneumothorax with collapsed upper lobe and normal lower lobe with no mediastinal shift (Figure 2). Subsequently, the patient was referred to the emergency department at the Al Shaab hospital for an urgent chest tube insertion. Upon admission, the patient's vitals were stable (HR: 20, RR: 26, BP: 140/90, and O_2_ saturation: 95% on room air). A chest tube was inserted, and the patient was started on low-flow oxygen via nasal prongs. Bedside echocardiography was performed; however, it was unremarkable apart from left ventricular concentric hypertrophy and consistent hypertensive heart disease with preserved left ventricular systolic function (EjFr: 64%) with grade one diastolic dysfunction.

**Figure 3:**
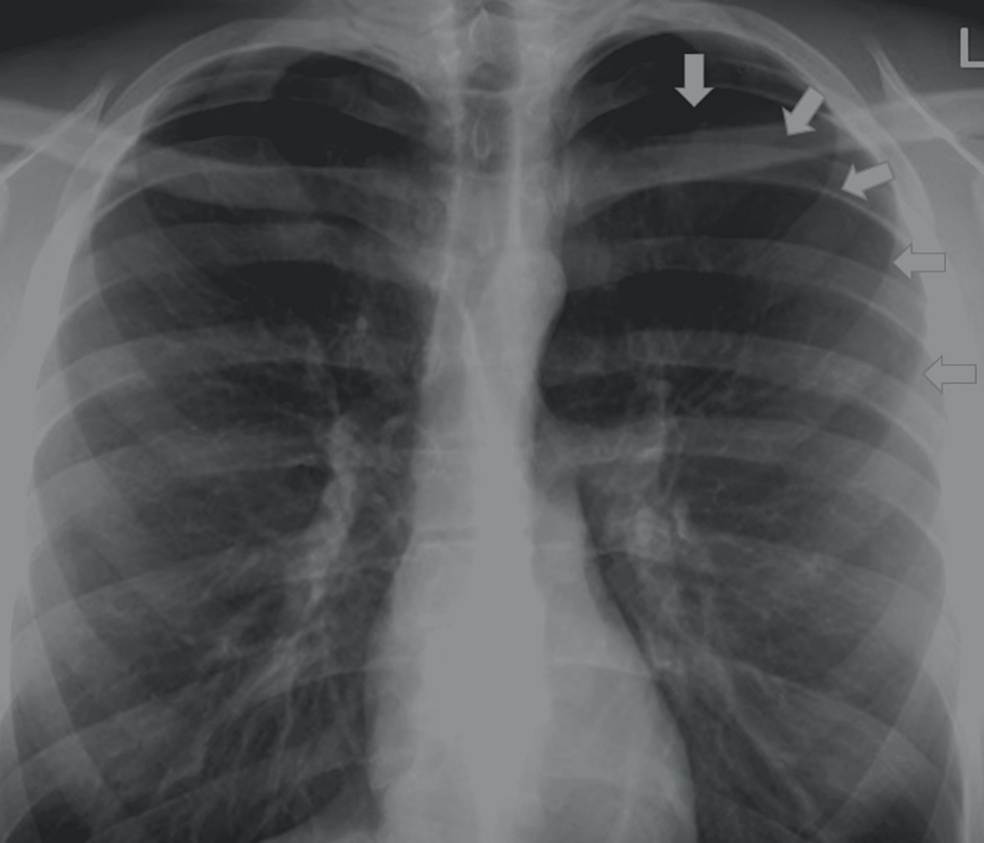
Recurrent massive left side pneumothorax (arrows)

Two days later, the patient's dyspnoea improved, and the chest tube was removed post full lung expansion/recovery, which was clinically and radiologically confirmed. The patient was discharged with a follow-up appointment after one week with a repeat chest X-ray (CXR). Although the patient was in good condition at her review appointment, being vitally stable with oxygen saturation of 96% on room air, her repeat CXR showed a recollapse of the left lung with pneumothorax (Figure 3). The patient was readmitted for chest drain reinsertion, revealing air bubbles but no fluid. She was started on high-flow oxygen (10 L/hour) and analgesia as per the hospital policy, with the oxygen saturation being maintained at 94%, and then tapered down over a few days. On day four of admission, the patient was taken off oxygen supplementation with a saturation of 96%. A tuberculosis Gene Xpert was requested, which returned negative, and all routine investigations [FBC, urea and electrolytes (U&E), and C-reactive protein (CRP)] were within normal ranges (Tables 1, 2).

**Table 1:** Routine blood workups RBCs: red blood cells; MCV: mean corpuscular volume; MCH: mean corpuscular hemoglobin; MCHC: mean corpuscular hemoglobin concentration; RDW: red cell distribution width; TWBCs: total white blood cells; ESR: erythrocyte sedimentation rate; CRP: C-reactive protein; TSH: thyroid-stimulating hormone

Test	Results	Normal range for an adult female
Hemoglobin	12.7 g/dL	12.0-16.0
RBCs	4.24 × 10^6^/µL	4.0-5.2
Hematocrit	38.1%	37-47
MCV	89.9 fL	76-96
MCH	30.0 pg	27-32
MCHC	33.3 g/dL	33-37
RDW	14.8%	11.5-14.5
Platelets	284 × 10^3^/µL	150-400
TWBCs	5.4 × 10^3^/µL	(4-11) × 10^3^/µL
Neutrophils	49.0%	40-70%
Lymphocytes	42.0%	20-45%
Monocytes	9%	2-8%
Basophils	0%	1-1%
Eosinophils	0%	1.2-1.6%
Creatinine	0.9 mg/dL	0.6-1.1
Blood urea	24.0 mg/dL	15-50
Sodium	137 mmol/L	135-150
Potassium	3.2 mmol/L	3.6-5.5
ESR	65 mm/hr	Up to 10
CRP	<3.0 mg/L	Negative: less than 10
TSH	1.5 mIU/mL	0.38-4.31
Free T3 (thyroxine)	3.3 pg/mL	2.17-3.34
Free T4 (thyroxine)	0.86 ng/dL	0.82-2.0

**Table 2:** Immunology workup ANA: antinuclear antibody; anti-dsDNA: anti-double-stranded DNA; anti-CCP: anti-cyclic citrullinated peptide antibody; anti-RNP: anti-ribonucleoprotein antibody; AMA: anti-mitochondrial antibody; anti-Scl-70: anti-scleroderma-70 antibodies; SmD1: Smith antibodies; anti-SS–A/Ro 60 KD: Sjogren anti-SS-A; anti-SS–A/Ro 52 KD: Sjogren anti-SS-B; anti-U1-snRNP: U1 small nuclear ribonucleoprotein particle

Test	Results	Normal range
ANA profile		
ANA global titer	1/640	>1/80
Pattern	Nucleolar + fine cytoplasmic granule	
ANA screening	0.4 NdX	Negative: <0.8, doubtful: 0.8-1.2, positive: >1.2
Anti-dsDNA	Negative	
Anti-SS–A/Ro 60 KD	Negative	
Anti-SS–A/Ro 52 KD	Negative	
Anti-Jo-1	Negative	
Anti-nucleosome	Negative	
Anti-histone	Negative	
Anti-SmD1	Negative	
Anti-PCNA	Negative	
Anti-PO	Negative	
Anti-CENPB	Negative	
Anti-Scl-70	Negative	
Anti-AMA-M2	Negative	
Anti-U1-snRNP	Negative	
Anti-SSB/La	Negative	
Anti-PM/Scl	Negative	
Anti-Mi-2	Negative	
Anti-Ku	Negative	
Rheumatoid factor	2.5 IU/mL	Negative: <12.0, doubtful: 12-18, positive: >18.0
Anti-CCP	2.4 U/mL	Negative: <12.0, doubtful: 12-18, positive: >18.0

On day seven of admission, the chest tube was still bubbling; 450 ml of clear fluid was drained and a surgical consultation was requested. Subsequently, a decision was made to perform thoracotomy with fistula repair due to persistent air leak. Intraoperatively, a ruptured infected single apical bulla was found on the left lung; consequently, a bullectomy with decortication was done, and the ruptured bullous sac was sent for histopathology. The sample revealed a fistula tract lined by columnar epithelial cells with adjoining fibrous connective tissue showing foci of heavy mixed inflammatory cellular infiltration and inflamed fibro-adipose tissue with no granuloma or malignancy, which is consistent with broncho-pleural fistula. Postoperatively, the patient recovered smoothly and was vitally stable with normal oxygen saturation on room air during her hospitalization period while on antibiotics and analgesia. She was discharged home on day eight in good condition and was followed up 10 days later in the clinic and was feeling well with normal observations. Also, her CXR showed complete recovery and no bullae. Further three-month follow-up was unremarkable with the patient feeling generally well and CXR revealing normal findings.

## Discussion

SS is one of the most common autoimmune diseases; it is characterized by the infiltration of various organs by CD4-T lymphocytes, with the lacrimal and salivary glands being the most often involved [5]. The disease primarily affects females (90%) and is associated with two periods of peak incidences: in the second/third decades of life and post-menopause, as reported in our case [6].

SS can be secondary to systemic lupus erythematosus (SLE), rheumatoid arthritis (RA), or scleroderma; therefore, clinical symptoms, blood investigation, and imaging are used to differentiate primary SS (pSS) from secondary SS (sSS) [5]. In our case, the absence of symptoms was suggestive of sSS, yet negative serum autoimmune antibodies supported a diagnosis of pSS.

Triple-negative variants account for 10% of pSS cases; nevertheless, the ANA titer is positive in 95% of cases. SS-A (52 kD, 60 KD) and SS-B (48 KD) are more specific to SS. Our patient had a high titer of ANA (1/640) but negative SS-A and SS-B; however, positive SS-A and SS-B were associated with early and severe disease [6].

Extraglandular manifestations of pSS affect 30-40% of the cases, mainly entailing neurological, renal, skin, and pulmonary involvement. Pulmonary manifestations are non-dominant and include xerotrachea, bronchiolitis, bronchiectasis, asthma, interstitial lung disease, nonspecific interstitial pneumonitis, lymphoid interstitial pneumonia, pulmonary cysts, and bronchus or lung-associated lymphomas. Respiratory manifestation may predate or coincide with the onset of the disease; additionally, pulmonary involvement in pSS is associated with high mortality [4-6]. Although the male gender is a risk factor for lung involvement in SS, clinically significant lung disease is predominantly found in females, consistent with our case [5]. Other risk factors for developing a pulmonary disease are smoking and late-onset pSS; however, our patient was an elderly nonsmoker [5,7].

Pulmonary involvement is associated chiefly with a positive anti-SSA, anti-SSB, and rheumatoid factor, which were negative in our patient; but ANA, which is proposed as a risk factor for pulmonary involvement, was positive [5]. Pleural involvement in immunological disease is infrequent with SLE, with RA representing the most common immunological disease with pleural involvement [8]. Pneumothorax as pleural manifestation is rare but reported in multiple cases.

In RA, pneumothorax, which is postulated to occur due to the rupture of a rheumatoid nodule, is rare but potentially resistant to standard care because immunosuppression predisposes to infection and poor healing. Additionally, surgical intervention is complex, and resistance to repeated talc pleurodesis has been reported [9]. SLE can also present with pneumothorax, which is extremely rare yet carries unfavorable outcomes, with a mortality rate in the reported cases reaching 33% and recurrence of 66%, which could be related to the rupture of the subpleural cavity triggered by lung infarction [10,11]. Pneumothorax has been reported in an extreme case of Wegener's with a poor outcome [12].

To the best of our knowledge, only two cases of pneumothorax associated with pSS have been reported [3,4]. Table 3 illustrates the reported cases' main findings. All reported cases were females, which shows that clinically significant pulmonary complication is more common in women. Furthermore, pneumothorax was the first presentation of pSS in two cases, including ours. Both the previously documented cases had bilateral diffuse cysts on the CT chest [3,4]; nonetheless, our patient had only a single apical bulla reported intraoperatively, and a ruptured subpleural cyst could have caused the pneumothorax development.

**Table 3:** Cases of primary Sjogren's syndrome with pneumothorax CT: computed tomography; ANA: antinuclear antibody; RF: rheumatoid factor; pSS: primary Sjogren's syndrome; SSA: Sjogren anti-SS-A; SSB: Sjogren anti-SS-B

Cases	Patient age/sex	Sjogren diagnosis	Affected side	Recurrence	CT finding	ANA profile	RF	Outcome
Watanabe et al. (2012) [3]	75 years/female	At pneumothorax onset	Right	No	Several bilateral thin-walled cysts	ANA, SSA (positive), SS-B -ve	-ve	Resolved after partial resection of the lung
Ismael et al. (2014) [4]	57 years/female	Long-standing pSS	Right	Yes	Large bilateral cyst	N/A	N/A	Death due to ventilator-associated pneumonia
Present case	60 years/female	At pneumothorax onset	Left	Yes	Left large pneumothorax, no cyst	ANA positive, SS-A/SS-B -ve	-ve	Full recovery after surgery

## Conclusions

Pneumothorax is an extremely rare but potentially unfavorable complication related to SS, with only two cases reported in the literature and usually associated with underlying lung pathology. Although infrequent, a prompt and clear approach toward diagnosis and management could positively impact the outcome and related comorbidities.
